# Detection of Atomic Scale Changes in the Free Volume Void Size of Three-Dimensional Colorectal Cancer Cell Culture Using Positron Annihilation Lifetime Spectroscopy

**DOI:** 10.1371/journal.pone.0083838

**Published:** 2014-01-02

**Authors:** Eneko Axpe, Tamara Lopez-Euba, Ainara Castellanos-Rubio, David Merida, Jose Angel Garcia, Leticia Plaza-Izurieta, Nora Fernandez-Jimenez, Fernando Plazaola, Jose Ramon Bilbao

**Affiliations:** 1 Department of Electricity and Electronics, University of the Basque Country - UPV/EHU, Leioa, Basque Country, Spain; 2 Immunogenetics Research Laboratory, BioCruces Research Institute, Barakaldo, Basque Country, Spain; 3 Department of Genetics, Physical Anthropology and Animal Physiology, University of the Basque Country - UPV/EHU, Leioa, Basque Country, Spain; 4 Department of Applied Physics II, University of the Basque Country - UPV/EHU, Leioa, Basque Country, Spain; Weizmann Institute of Science, Israel

## Abstract

Positron annihilation lifetime spectroscopy (PALS) provides a direct measurement of the free volume void sizes in polymers and biological systems. This free volume is critical in explaining and understanding physical and mechanical properties of polymers. Moreover, PALS has been recently proposed as a potential tool in detecting cancer at early stages, probing the differences in the subnanometer scale free volume voids between cancerous/healthy skin samples of the same patient. Despite several investigations on free volume in complex cancerous tissues, no positron annihilation studies of living cancer cell cultures have been reported. We demonstrate that PALS can be applied to the study in human living 3D cell cultures. The technique is also capable to detect atomic scale changes in the size of the free volume voids due to the biological responses to TGF-β. PALS may be developed to characterize the effect of different culture conditions in the free volume voids of cells grown *in vitro*.

## Introduction

Free volume voids play a key role in a variety of mechanical properties in polymers [1] and dynamic processes in biological systems, including permeability of small molecules and diffusion of drugs through cell membranes [2]. Positron annihilation lifetime spectroscopy (PALS) provides a direct measurement of the free volume void sizes in polymers and biological systems at the molecular level, and this free volume is critical to understand physical and mechanical properties of complex biomolecular systems as collagen [3], porcine eye lens [4], rat brain sections [5] and human skin [6].

It has been recently shown that PALS is sensitive to changes in the free volume voids size that are associated with certain types of tumors and is able to discriminate between healthy and cancerous skin samples from the same patient. Thus, PALS has been proposed as a potential tool to detect cancer formation at the early stages of tumor development [6], [7]. However, cellular composition of tissue samples varies from the same organism and may involve changes in the void sizes [8].

Our hypothesis is that biological responses to environmental factors, such as cellular differentiation and changes in cell colony structure are accompanied by atomic scale changes in the free volume void size of the cells, and that those changes can be characterized by PALS. To test this idea, we used a T84 human colon cancer 3D cell culture model under two different conditions that result in distinct *in vitro* multicellular organization of colonies. Cells were grown within a type I collagen gel matrix with or without transforming growth factor β (TGF-β), a fibroblast-derived factor affecting epithelial cell proliferation, differentiation, motility, and T84 cell differentiation. Cells in collagen form unorganized 3D cell clusters within the gels, but when grown in the presence of TGF-β, T84 epithelial cells are able to organize and differentiate into a distinct phenotype that resembles intestinal crypts [9]. We show that PALS measurements are able to detect atomic scale changes of voids size in human colonic adenocarcinoma 3D cell cultures developing over time and when treated with TGF-β.

## Materials and Methods

### Cell culture

Human colon adenocarcinoma T84 cell line (CCL 248, ATCC Rockville, MD, USA) was kindly provided by Prof. Markku Maki (Celiac Disease Study Group, University of Tampere, Finland). Cells were maintained in culture in the laboratory. Dulbecco's modified Eagle's medium F12 nutrient mixture (1∶1) (DMEM-F12 ref 31330), penicillin-streptomycin (ref 15070) and sodium bicarbonate were purchased from Life Technologies (Spain). Heat inactivated foetal bovine serum (ref F9665), 10× RPMI-1640 (ref R1145), trypsin/EDTA (ref T4049), Triton X-100 (ref T9284), albumim from bovine serum (ref B4287) and ribonuclease A (ref R6513) were obtained from Sigma-Aldrich (Spain). Rat tail Collagen I (ref 354236) was purchased from Beckton Dickinson (Spain). Transforming growth factor beta 1 recombinant human (rhTGF-β, ref 240-B) was purchased from R&D systems (United Kingdom) and paraformaldehyde (ref 15710) from electron microscopy sciences, Hoetch 33342 and Phalloidin-PE were obtained from Sigma-Aldrich (Spain). Plastic dishes were obtained from Corning Costar (Spain) and plates from Becton Dickinson (Spain).

Cells were maintained in culture medium composed of DMEM-F12, 5% heat inactivated foetal bovine serum and 1% penicillin-streptomycin [9]. T84 cells were grown on T75 tissue culture flasks at 37°C in a 5% CO_2_ atmosphere and passaged once a week upon reaching 80% confluence. Culture medium was changed every 2 days. T84 cells were removed from the flasks with 6 ml 0,25% trypsin-EDTA at 37°C during 15 minutes. Then, were washed in medium, collected by centrifugation at 450xg during 5 minutes and subcultured in 24-well plates with collagen. Cells in suspension were counted with a cellular counter (*Coulter particle count and size analyser*, Beckman Coulter). T84 cells were plated in 24 well plates at a density of 1.5×10^5^ cells/well.

For generating 3-D aggregates rat tail Collagen I was used. Collagen was mixed with acetic acid, 10× RPMI-1640 and sodium bicarbonate. In each experiment we used 1 ml collagen, 125 µl RPMI-1640 10× and 125 µl sodium bicarbonate.

To create the 3D culture, 300 µl of the collagen mix (3 mg/ml final concentration) were added to each well and incubated at 37°C to gelify. After 20 minutes of incubation, cells were resuspended in additional 200 µl of the collagen mix and were seeded on collagen gels. Finally, 1 ml DMEM-F12 culture medium was added to cover 3D cultures.

To determine the optimal length of the experiments (i.e. the suitable time in the cell cultures to measure their mean free volume through PALS) we have chosen different culture times to make measurements: 6-13-20-27-34-41 days.

### Cell stimulation

To induce changes in cell morphology, T84 aggregates were incubated with 10 ng/ml hrTGF-β1 added to the culture medium [9]. These cultures were grown in culture until 41 days and the culture medium was changed every two days.

### Immunofluorescence microscopy

7 and 17 day-old 3D cell cultures were fixed with 4% paraformaldehyde at room temperature for 30 minutes. After washing three times with PBS, cultures were permeabilized with 0.5% Triton X-100 at 4°C overnight and washed again with PBS. Following blocking with 3% BSA in PBS at room temperature during 2 hours, cultures were incubated with RnaseA 1× (1∶1000) at room temperature for 1 hour [10]. Filamentous actin was labeled with phalloidin-PE (1∶1000) at 4°C overnight [11]. Cell nuclei were stained with Hoechst 33342 (1∶1000) at 4°C for 3 hours.

Samples were analyzed with an Olympus Fluoview FV500 confocal microscope. Stack deconvolution and 3D reconstruction of images taken on the z axis to construct motion videos were performed with ImageJ software.

### Free volume void size measurements

With respect to the spectrometer, ORTEC (USA) electronic modules were equipped with two BC-422 plastic scintillators from Saint Gobain (USA) and two H1949-50 Hamamatsu (Japan) photo multipliers suited in vertical position inside of a FFD-1402 refrigerator made by Radiber S.A. (Spain). The positron source was ^22^NaCl from PerkinElmer (USA) of about 15 µCi, evaporated between two Kapton foils of 7.5 µm from CS Hyde (USA) and of 75 µm from DuPont (USA) sealed by a double sided Kapton tape from CAPLINQ (Canada). Since we intended to measure fresh, living cell cultures, we designed and fabricated a specific sample holder for the study (supplementary fig. 1).

**Figure 1:**
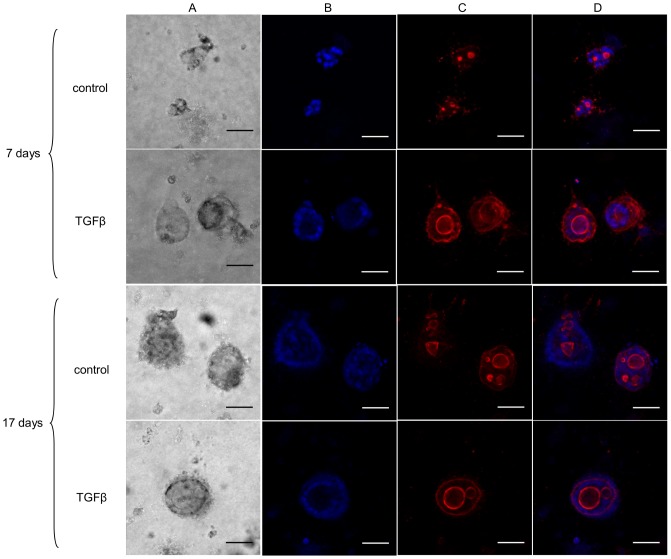
Effect of TGF-β on T84 3D epithelial cell organization. Confocal microscopy of 7 and 17 day-old T84 in three-dimensional culture. All images are at 20× magnification. Scale bar represent 50 µm. A) T84 colonies are observed with phase contrast. B) Cell nuclei stained with Hoechst. C) Filamentous actin is stained with Phalloidin-PE. In D, merged images of B and C.

PALS experiments were performed by a fast-fast timing coincidence system with a full width at half maximum (FWHM) resolution of 0.260 ns. The measurements were conducted at 4°C. The lifetime of positrons remained unchanged along the time by the exposition to the source radiation during three measurements. The lifetime spectra collected with more than 3×10^6^ counts per spectrum were analyzed by using the LT_polymers program [12]. After subtracting the source contribution (31.6%, 0.382 ns), positron lifetimes spectra were decomposed into three components. The longest-lived component distribution was assigned to the *ortho*-Positronium (*o*-Ps) lifetime distribution as previously described [6]. The average radius size of the voids volume in 3D cultures were estimated via the Tao-Eldrup equation [13], [14], based on positronium trapping in spherical voids in polymers:
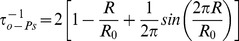
where *R_0_  =  R +* Δ*R*, and Δ*R* is an empirical parameter fitted to be 1.66 Å [15].

## Results

T84 intestinal epithelial cells grown three dimensionally formed unorganized round colonies in 7 and 17 day-old cultures (Figure 1. control). Cells were unpolarized and gathered around in globule clusters without organized orientation. Cell nuclei were distributed around the colony but in an unorganized manner (Video S1, Video S2). However, when T84 cells were grown three dimensionally in the presence of TGF -β, cell colonies presented a highly organized structure, as a one-cell layer around a lumen (Figure 1. TGF-β, Video S3, Video S4) surrounding a central lumen. Filamentous actin distribution (Figure 1.C) localized in the lining of the sphere lumens, reflecting TGF-β mediated differentiation (Video S3, Video S4). This highly organized structure is well perceived by several videos in the supplementary information.

**Figure 2:**
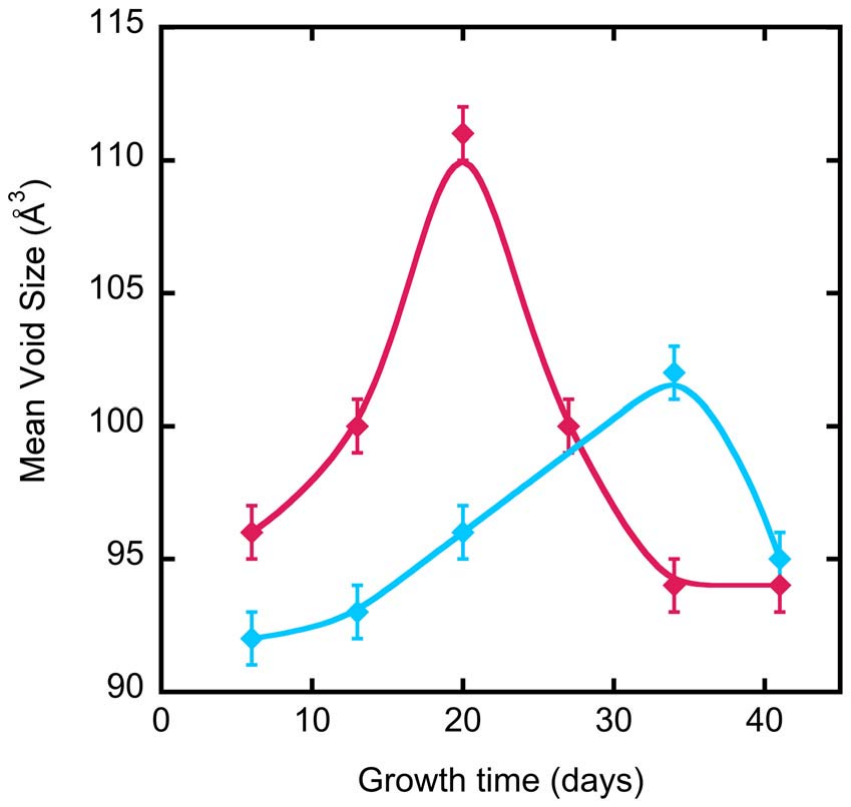
Growth time dependence of mean void volume in human colonic adenocarcinoma T84 3D cell cultures. The blue curve represents control cultures and the red curve represents cultures treated with TGF-β.

Data obtained by PALS are summarized in Table 1. In terms of free volume void sizes, in both unstimulated and TGF-β growth conditions the void volumes of the 3D cultures increased to reach a maximum (Figure 2); For normal growth conditions the mean void volume in the peak was 102±1 Å^3^ at 34 days, while for the culture with TGF-β was 111±1 Å^3^ at 20 days. As shown in Figure 2, once the mean void size reached a maximun, this value started to decrease gradually: the mean void volume decreased 7% under normal conditions in 1 week, and 16% in 3 weeks under TGF-β conditions.

**Table 1:** *o*-Ps mean lifetime (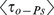, ns) and distribution (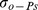, ns) in collagen I and T84 human colonic adenocarcinoma three-dimensional cell cultures grown in normal conditions and with TGF-β, at different growth time points.

	Collagen I		Normal medium		Supplemented with TGF-β	
Growth time (days)	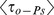 (ns)	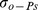 (ns)	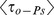 (ns)	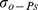 (ns)	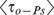 (ns)	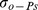 (ns)
**	1.92±0.02	0.20±0.04	-	-	-	-
6	-	-	1.95±0.03	0.28±0.05	1.99±0.02	0.39±0.03
13	-	-	1.96±0.01	0.29±0.02	2.03±0.03	0.55±0.05
20	-	-	1.99±0.03	0.24±0.06	2.15±0.01	0.47±0.01
27	-	-	-	-	2.03±0.02	0.42±0.03
34	-	-	2.06±0.01	0.37±0.01	1.97±0.03	0.48±0.04
41	-	-	1.98±0.02	0.30±0.04	1.97±0.03	0.44±0.04

Collagen I is the matrix of all three-dimension cultures measured during the growth time.

The mean lifetime and distribution *o*-Ps in three-dimensional cell cultures were respectively 2–12% and 20–120% larger than in collagen (Table 1). Depending on the growth time point, the *o*-Ps lifetime distribution for TGF-β cultures was between 0.11±0.08 ns to 0.26±0.07 ns larger than under unstimulated growth conditions.

## Discussion

We have shown that the T84 3D culture model allows the specific study of free volume in living cells by PALS. We found that PALS detects atomic scale changes of the mean free-volume void size in human colonic adenocarcinoma 3D cell cultures over time. Morphological changes observed by immunofluorescence microscopy in cell cultures in different conditions are surely the consequence of molecular events that are taking place in the cells and are also reflected in PALS measurements. Although cell culture developmental events can not be correlated directly to the free volume, results suggest that cellular changes are indeed reflected in the nanostructure of these cultures. The *o*-Ps lifetime changes probably emerge from the dynamic processes of cell cultures (growth, division and death). Furthermore, TGF-β induces cell differentation, and this is acompained by an increase in the distribution of *o*-Ps lifetime (and thus, of free volume size) in the 3D cell cultures. This result implies a greater dispersion of hole size in samples treated with TGF-β compared to unstimulated conditions. Cell death could be involved in the drop in the *o*-Ps lifetimes.

We are at the dawn of PALS applications in complex cell systems, but our results show that 3D cell cultures (*o*-Ps lifetime is measured in a mixture of cells and collagen) can be a good starting point for this type of research. Currently, PALS only allows bulk measurements that do not provide sufficient resolution to distinguish *o*-Ps lifetimes in individual cells from the whole culture. Nonetheless, the measured increase in lifetimes and distributions must be due to molecular and structural changes that affect the free volume of the cells and consequently that of the 3D culture. Furthermore, PALS is a non-perturbative technique; it perturbs neither the structure of the 3D culture nor cell dynamics. We used the Tao-Eldrup model for polymers when calculating the sizes of free volume voids from the *o*-Ps lifetime, as previously done in many PALS studies in biological systems [3]–[7].

To the best of our knowledge, this is the first study of the application of PALS in 3D living cell cultures and indeed, more measurements in different cell lines and culture conditions that modify different biological parameters are needed before this method can be considered a potentially useful diagnostic tool in human disease. Together with the experimental research, studies to show whether the local electric fields caused by transmembrane voltage of the cells are affecting the positron signal, or which parts of the cell are preferred targets for the positron annihilation should be also undertaken. Additionally, computational simulations to evaluate the fit of the Tao-Eldrup model (implemented for free-volume characterization in polymers) into more complex biological matter will be necessary.

In conclusion, this is the first study applying PALS for the characterization of living cells. Our results demonstrate that the 3D cell cultures are useful for measuring free volume in living cells by PALS in a realistic and controlled way, and for the observation of changes in free volume void sizes in cell cultures due to specific factors (e.g., growth time and TGF-β). This study is in agreement with PALS technique as a valid tool for the characterization of biological systems and could provide useful information in cancer research at the atomic and molecular scale.

## Supporting Information

Figure S1 **Design and setup of the sample holder and positron source for the application of PALS in biological samples and liquids.** We designed and fabricated an aluminum sample holder for measuring biological samples and liquids. The scintillators and photomultipliers used in this work were placed in a vertical position. The figure shows the longitudinal section of the sample holder (1). In (2) we describe the sample filling the lower side of the holder. In (3), we position the kapton source over the sample, sitting on the aluminum projection. We can also see how the ^22^Na is previously deposited just in the middle of the kapton, so all the positrons annihilate in the sample, and not in the holder. This kapton source needs to be robust, so we used a thicker kapton foil of 75 µm for the part sitting on the holder projection (Kapton foil B), compared to the 7.5-µm kapton foil A. In (4) we show how the positron source is sandwiched by two identical liquid/biological samples.
(TIFF)

Video S1 **Confocal immunofluorescence of three-dimensional T84 colonies after 7 days in culture.**
(AVI)

Video S2 **Confocal immunofluorescence of three-dimensional T84 colonies after 17 days in culture.**
(AVI)

Video S3 **Confocal immunofluorescence of three-dimensional T84 colonies after 7 days in culture with 10 ng/ml TGF-β.**
(AVI)

Video S4 **Confocal immunofluorescence of three-dimensional T84 colonies after 17 days in culture with 10 ng/ml TGF-β.**
(AVI)
