# Supplementary figures and images for: Detection of Atomic Scale Changes in the Free Volume Void Size of Three-Dimensional Colorectal Cancer Cell Culture Using Positron Annihilation Lifetime Spectroscopy
Source: PLoS One. 2014 Jan 2;9(1):e83838. doi: 10.1371/journal.pone.0083838 (PMC3879280; doi:10.1371/journal.pone.0083838)

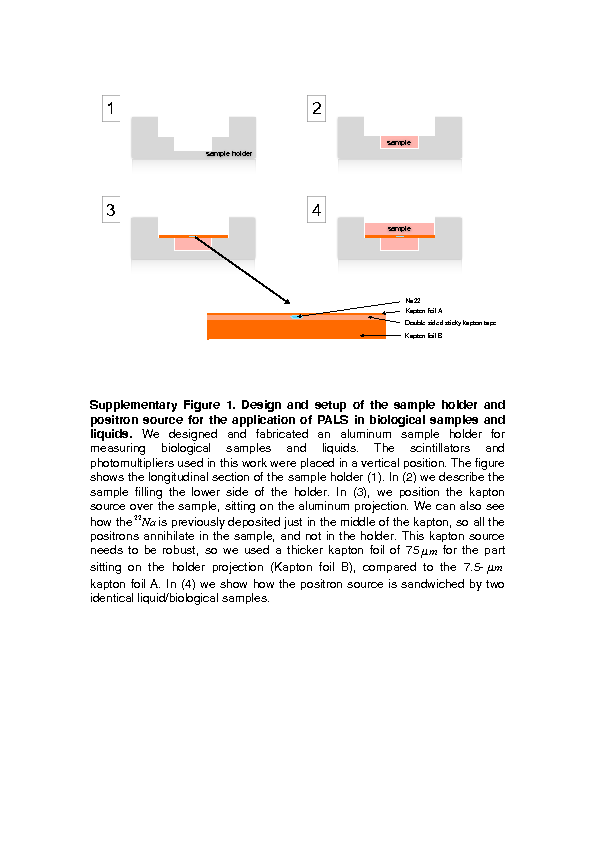

Supplement: Figure S1 — Design and setup of the sample holder and positron source for the application of PALS in biological samples and liquids. We designed and fabricated an aluminum sample holder for measuring biological samples and liquids. The scintillators and photomultipliers used in this work were placed in a vertical position. The figure shows the longitudinal section of the sample holder (1). In (2) we describe the sample filling the lower side of the holder. In (3), we position the kapton source over the sample, sitting on the aluminum projection. We can also see how the 22Na is previously deposited just in the middle of the kapton, so all the positrons annihilate in the sample, and not in the holder. This kapton source needs to be robust, so we used a thicker kapton foil of 75 µm for the part sitting on the holder projection (Kapton foil B), compared to the 7.5-µm kapton foil A. In (4) we show how the positron source is sandwiched by two identical liquid/biological samples. (TIFF) [file pone.0083838.s001.tiff]
